# Human Cortex Spheroid with a Functional Blood Brain Barrier for High-Throughput Neurotoxicity Screening and Disease Modeling

**DOI:** 10.1038/s41598-018-25603-5

**Published:** 2018-05-09

**Authors:** Goodwell Nzou, R. T. Wicks, E. E. Wicks, S. A. Seale, C. H. Sane, A. Chen, S. V. Murphy, J. D. Jackson, A. J. Atala

**Affiliations:** 1 Wake Forest Institute for Regenerative Medicine, Wake Forest School of Medicine, Winston-Salem, NC 27101 USA; 2 Department of Neurological Surgery, Wake Forest Baptist Medical Center, Winston-Salem, NC 27157 USA

## Abstract

The integral selectivity characteristic of the blood brain barrier (BBB) limits therapeutic options for many neurologic diseases and disorders. Currently, very little is known about the mechanisms that govern the dynamic nature of the BBB. Recent reports have focused on the development and application of human brain organoids developed from neuro-progenitor cells. While these models provide an excellent platform to study the effects of disease and genetic aberrances on brain development, they may not model the microvasculature and BBB of the adult human cortex. To date, most *in vitro* BBB models utilize endothelial cells, pericytes and astrocytes. We report a 3D spheroid model of the BBB comprising all major cell types, including neurons, microglia and oligodendrocytes, to recapitulate more closely normal human brain tissue. Spheroids show expression of tight junctions, adherens junctions, adherens junction-associated proteins and cell specific markers. Functional assessment using MPTP, MPP+ and mercury chloride indicate charge selectivity through the barrier. Junctional protein distribution was altered under hypoxic conditions. Our spheroid model may have potential applications in drug discovery, disease modeling, neurotoxicity and cytotoxicity testing.

## Introduction

Over a million adults are annually diagnosed with brain diseases or disorders in the US alone^1^. The Alzheimer’s Association reported that in 2016, the national cost for dementias was approximately $236 billion^2^. A major contributor to this high treatment cost is the late stage failure of promising drug candidates. Only 8% of central nervous system drug candidates that reach initial Phase 1 human safety testing eventually achieve regulatory approval due to either toxicity or ineffectiveness^3^. This estimate does not include the number of initially promising treatments pursued during pre-clinical *in vitro* and *in vivo* studies^3^. The shortage of effective therapies and low success rate of investigational drugs are in part due to the lack of human equivalent models^4^. Some current 2D systems may not accurately mimic human physiology because they do not possess the three dimensional organization of tissues, and often consist of cell lines, lacking physiologically relevant ratios of all cell types present in the organ^5,6^. More recently established 3D blood brain barrier (BBB) models have provided an understanding of size exclusion, selectivity and many other important aspects such as the expression of efflux and transport of proteins. Urich *et al*. reported the successful assembly of human primary astrocytes, pericytes, and endothelial cells into a BBB− like model^7^. Cho *et al*. went on to characterize the BBB properties of a similar model^8^.

The formation, maintenance and function of the BBB in the human neurovascular unit (NVU) depend on highly specific intercellular interactions within the human brain: specifically, the association between pericytes and blood vessels and how they regulate human brain endothelial cell proliferation, survival, migration, differentiation, and vascular branching^9^. This signifies direct pericyte-endothelial interactions in the lumen and their crucial role in maintaining the integrity of the BBB in the human neurovascular unit^10,11^. The interaction between astrocytes and oligodendrocytes and the direct influence of astrocyte on the integrity of the BBB are also well documented^12–15^, There is a significant body of evidence, in both *in vitro* and *in vivo* systems, highlighting astrocyte interactions with the cerebral endothelium and the ways in which they help determine BBB function, morphology, and protein expression^16–19^. Microglia also play a critical role in BBB regulation and modulation of tight junction expression. They in turn have complex integrations in brain diseases such as epilepsy, ischemic stroke, and neurodegenerative disorders^20^. Furthermore, neurons are known to induce BBB related enzymes^21^. Therefore, the inclusion of the major human NVU cell types in an *in vitro* organoid model would be helpful in predicting human physiologic conditions.

A natural progression from the currently established BBB models would be to define the effect of chemical agents on all the cell types that are critical to the normal function of a human NVU, including the microglia, oligodendrocytes and neurons that are adjacent to the BBB, and to further understand the intercellular dynamics once molecules cross the barrier. Therefore, the development of a 3D *in vitro* system that contains all major cell types found in adult human brain cortex may provide a platform that can be used to understand the fundamental principles at play with the BBB, its function, and also to understand the effects of chemical substances that cross the BBB.

Here we report the development of a human neurovascular unit organoid model that contains the six constituent cell types found within the brain cortex: human brain microvascular endothelial cells (HBMEC), human pericytes (HBVP), human astrocytes (HA), human microglia (HM), human oligodendrocytes (HO) and human neurons (HN), with endothelial cells enclosing the brain parenchymal cells. Cells derived from induced pluripotent stem cell (iPSC) sources (HM, HO, and HN) were utilized in several instances to establish potential patient-specific and disease applications. We validated the expression of tight junctions, adherens junctions and transport proteins, and showed that this model can be used in toxicity assessment studies for molecules that have the potential to cross or open the BBB. Neurotoxicity screening was assessed with the effects of mercury chloride, MPTP and MPP+. In addition, a model of the blood brain barrier during clinical ischemia was established showing physiologic responses under hypoxic conditions.

## Results

### Confirmation of Assembly with Barrier Formation

**Figure 1:**
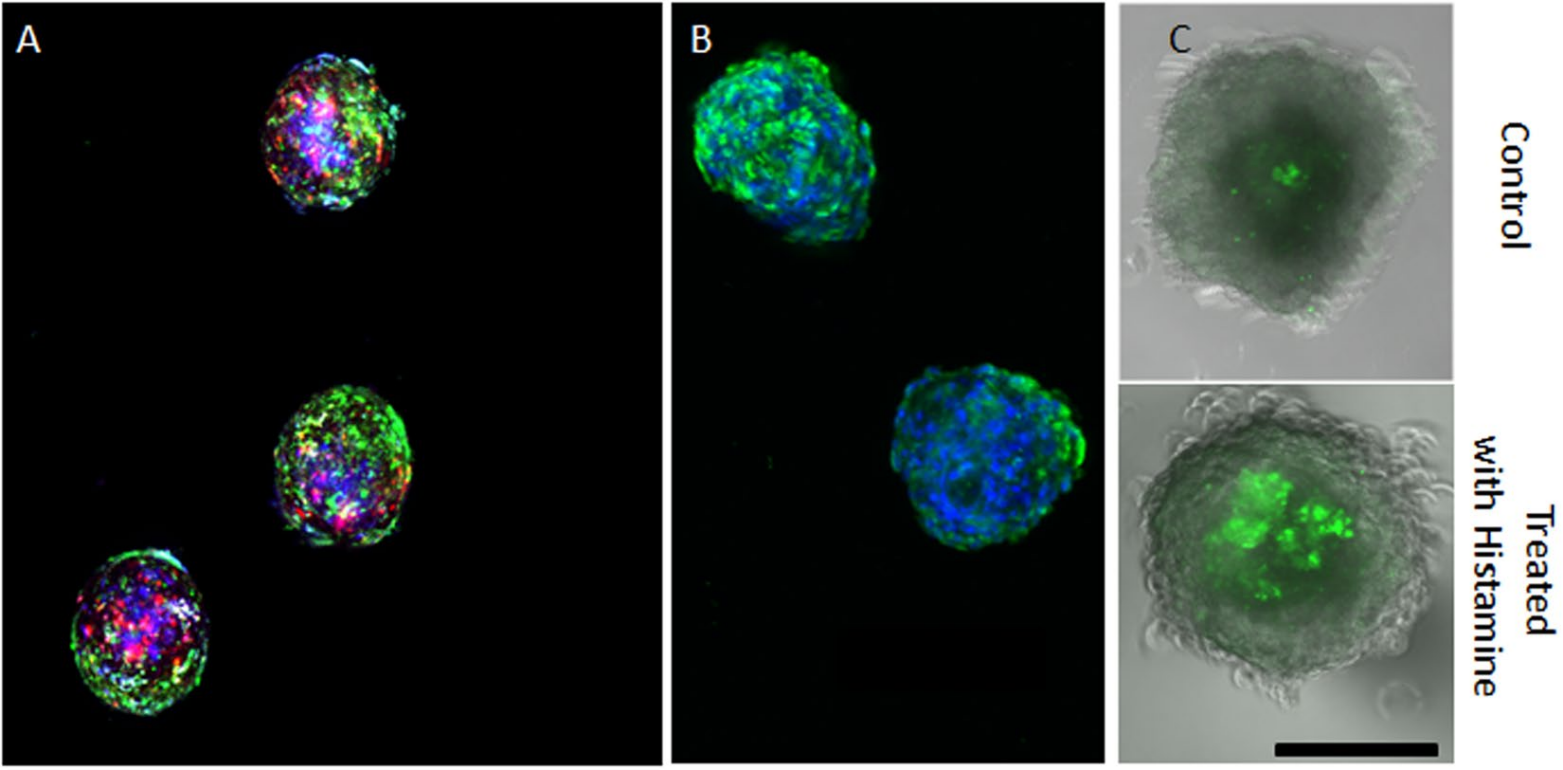
Assessment of cell localization, tight junction protein expression, and barrier properties in self-assembled spheroids. Spheroids in (**A**) show self organisation of endothelial cells and pericytes to the outermost sphere whereas the astrocytes and neurons (violet) are encapsulated by the endothelial cells and pericytes. (**B**) Spheroids containing HBMEC, astrocytes, and pericytes were formed through self assembly. Shown is ZO-1, a tight junction protein that prevents free paracellular transport of molecules across the vascular-type barrier. Dapi (blue) shows nuclear staining. Also shown in the figure are empty pockets where there is no ZO-1, this indicates that the HBMECs are likely not completely covering the spheroid. (**C**) Permeability assessment was done through incubating the spheroids containing HBMEC, pericytes and astrocytes with FITC labeled IgG. The upper panel represents spheroids with an uncompromised vascular-type barrier (control) and the lower panel shows spheroids that were treated with histamine to transiently open the vascular-type barrier. Scale bar, 300 μm.

Prior publications have shown the assembly of three cell types: primary human astrocytes, human pericytes, and human brain microvascular endothelial cells^7^. To demonstrate the assembly and cellular organization of organoids with four cell types, HBMECs, HPs, HAs, and human neuronal cells (HCN-2) were pre-treated with long-term cell labeling dye. The four cell types were placed into a mixture containing a ratio of 1:1:5:6, respectively. The cell mixture was placed in a hanging drop culture environment. After 96 hrs of culture, assembled organoids were lowered and confocal microscopy images confirmed HBMEC and HBVP localization to the periphery of the organoid, with HA localized near the surface, and neuronal cells forming the core (Fig. 1A). We evaluated the presence of a BBB in our organoid model by testing the expression of BBB tight junction protein ZO-1, which was identified with immunofluorescent labelling and confocal microscopy (Fig. 1B).

We characterized the integrity and dynamic properties of the barrier formed in the organoids containing HBMEC, HBVP and HA by assessing FITC-labelled IgG permeability across the BBB. IgG permeability was first evaluated in untreated organoids. We then assessed IgG permeability in organoids pretreated with histamine. Histamine is a chemical agent known to transiently open the BBB^22–24^. Qualitative analysis through confocal microscopy showed higher FITC fluorescence at the core of treated organoids (Fig. 1C) compared to untreated organoids (Fig. 1C). This experiment shows that a type of vascular barrier was present. In order to specify BBB characteristics, we conducted further experiments using a small molecule as will be discussed below. A small amount of IgG was able to be visualized within the three cell-type organoid core in untreated organoids. The effects observed here could be attributed to incomplete coverage of endothelial cells.

### Six Cell Type Human Cortical Organoid

**Figure 2:**
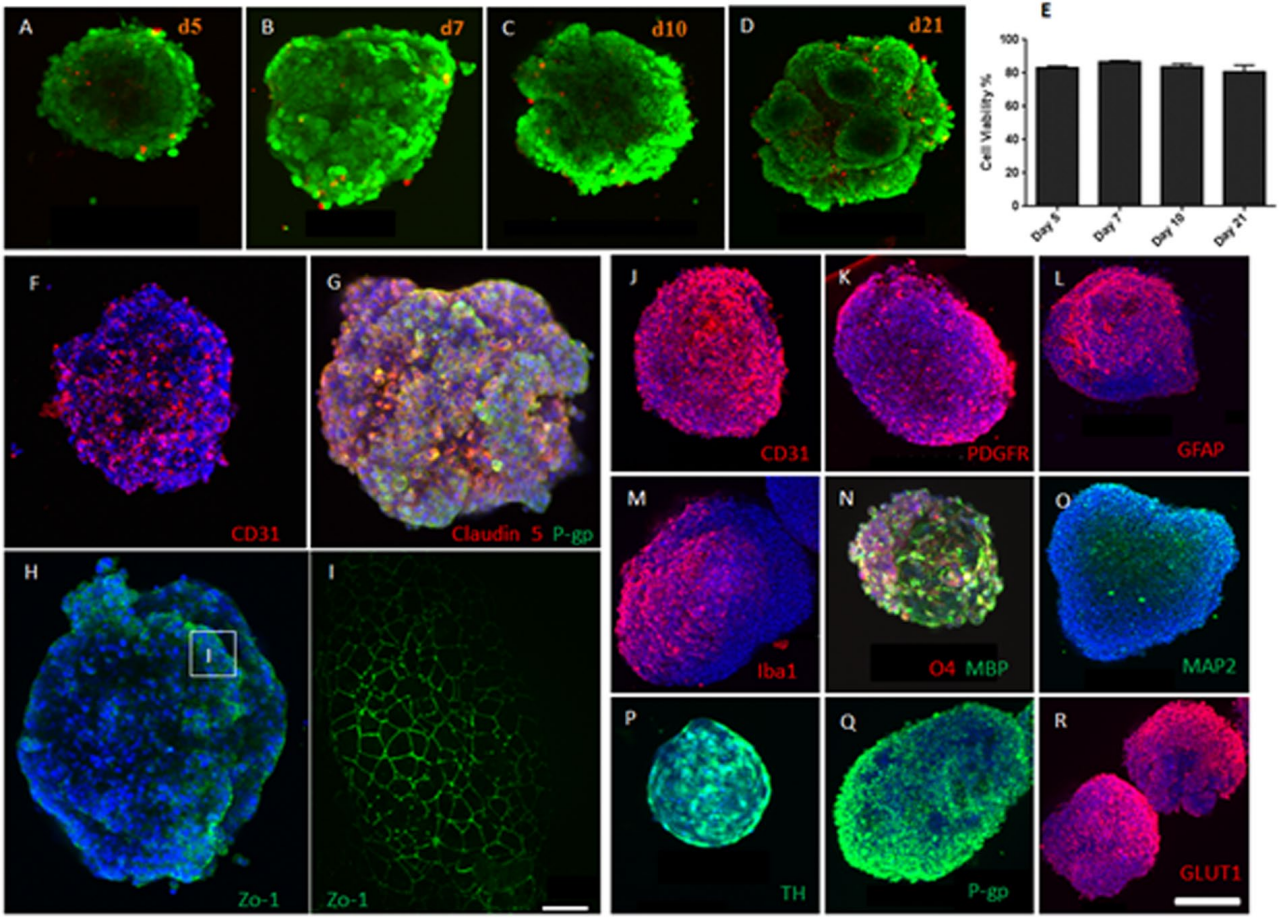
Six cell-type spheroid Characterization. Live-Dead Staining was conducted on days 5 (**A**), 7 (**B**), 10 (**C**), and 21 (**D**) to assess cell viability. Green represents live cells and red indicates dead cells; In each case, 8 randomly selected spheroids were assed for cell viability. (**E**) Green florescence for live cells was quantified and expressed as a percentage of the total fluorescence (red and green). Immunohistochemistry was conducted on spheroids to determine phenotypic protein expression. CD31 expression (**F**) shows localization of HBMEC in self-assembled spheroids. Even though the cells migrate to the outer sphere, the endothelial cells do not cover the spheroid. Expression of claudin-5 (**G**, red) was established through immunofluorescent staining. Expression of P-gp in **G** and **Q** (an efflux protein responsible for the efflux of xenobiotic substances out of the brain tissue) was established in self –assembly and staged self-assembly spheroids, respectively. Self-assembly spheroids were stained for ZO-1 (**H**) and DAPI (blue). Selected area was imaged at higher magnification in order to visualize the cell border connections formed by ZO-1 (**I**). Specific cell markers were used to identify normal protein expression in individual cell type incorporated into the spheroid made through a staged self-assembly: (**J**) showing CD31 staining signifying that the HBMEC cover the spheroid in a staged self-assembly, PDGFR (**K**) an pericyte marker, GFAP (**L**) an astrocyte marker, Iba1 (**M**) a microglial marker, **N** O4 and MBP markers of oligodendrocyte progenitors and mature oligodendrocytes respectively. MAP2 (**O**) is a neuronal marker. Neuronal spheroids were stained for Tyrosine Hydroxylase (**P**) to identify the presence of dopaminergic neurons. Transmembrane glucose transporter and efflux transporter protein expression and localization were established by staining for P-gp (**Q**) and GLUT1 (**R**) respectively. All markers were found to have a thorough distribution. Images were obtained at 10x magnification. Scale bars are depicted in white. Scale bars, 200 μm and 30 μm (for L only).

We constructed organoids containing HBMECs, HBVP, HA, HM, HO and HN using a single self-assembly method to assess the interaction of all 6 cell types. The organoids maintained very high cell viability for up to 21 days *in vitro* (Fig. 2A–E). Cell viability, was uniform in all Z-stacked slices utilized in quantitative data presented. There were no apparent differences observed between the core and the outer surface as visualized through confocal microscopy. Immunofluorescent staining for the HBMEC marker CD31 to identify endothelial cell coverage in these assembled organoids showed that the endothelial cells localized to the outer surface of the organoid (Fig. 2F). We identified the expression of tight junction proteins: ZO-1 and Claudin-5 (Fig. 2G–I). The endothelial cells, however, did not fully cover the outer surface of the organoids. This is shown with sections on organoids not staining for tight junction proteins ZO-1, and claudin-5, and CD31 (Fig. 2F–H). In order to increase endothelial coverage on the surface of the organoids, we utilized a staging method of organoid assembly. HBMVECs and HBVP were added 48 hrs after forming organoids with the 4 cell types (HA, HM, HO, and HN), which allowed for a more cohesive coating of the organoid surface. This method is henceforth designated as the staged assembly method.

### Organoid Characterization

**Figure 3:**
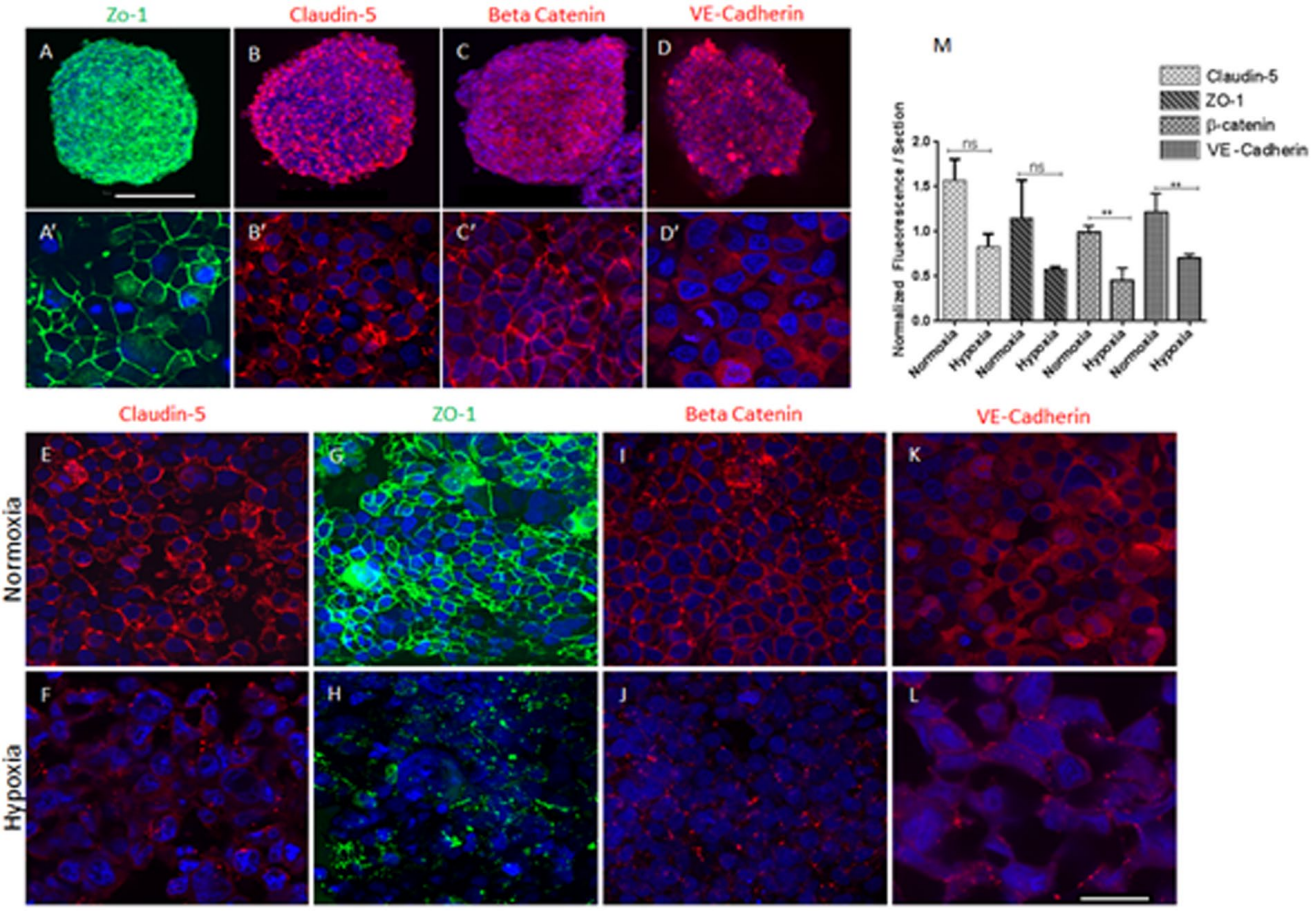
Blood-brain barrier marker expression and effect of hypoxia on tight and adherens junction protein distribution. The presence of tight junction markers Zona occludens-1 (ZO-1) (**A**), claudin-5 (**B**), and adherens junction proteins β-catenin (**C**) and VE-Cadherin (**D**) were confirmed via immunofluorescent staining and confocal microscopy at 10x. The lower panels in A′–D′ are parts of spheroids in A–D taken at higher magnification. Immunofluorescent staining was conducted on spheroids consisting of all types for junction markers claudin-5, ZO-1, β-catenin, and Ve- cadherin under normoxic (upper panel, E, G, I, & **K**). The lower panels in (**F**, **H**, **J**, & **L**) show spheroids that were cultured under hypoxic conditions at 37 °C, 0.1% O_2_ for 24 hrs prior to immunofluorescent staining and imaging. Spheroids under hypoxic condition exhibited disrupted intercellular junction markers compared to their normoxic counterparts. Images were taken at higher magnification. (**M**) Fluorescence for claudin-5, ZO-1, β-catenin and VE-cadherin was quantified and normalized to DAPI fluorescence per section. Student T-Test, two tailed hypothesis **P $<$ 0.05 with normoxic condition, n_organoids_ = 3. Data represented as mean ± s.e.m. Scale bars 250 μm (**A**–**D**) and 50 μm (**E**–**L**).

Organoids were constructed using the staged assembly method and stained positively for cell specific markers, CD 31 for HBMVEC (Fig. 2J), platelet-derived growth factor receptor-beta (PDGFR) for pericytes^25^ (Fig. 2K), glial fibrillary acidic protein (GFAP), marker for astrocytes (Fig. 2L)^26^, ionized calcium-binding adapter molecule 1 (Iba1) for microglia^27^ (Fig. 2M), myelin basic protein (MBP) and O4 for oligodendrocytes^28^ (Fig. 2N), and MAP2 and tyrosine hydroxylase (TH) for mature and dopaminergic neurons (Fig. 2O and P), respectively. TH staining performed on neuronal spheroids may overstate the proportion of TH+ neurons in assembled organoids with all cell types. The proportion of TH+ neurons in organoids containing all cell types could be inferred from the MAP2 positive cells in 6 cell type organoid showing the presence of mature neurons. Together, these cells act in concert to enhance the function of the BBB, thereby maintaining brain homeostasis in humans^29^. Permeability –glycoprotein (P-gp; Fig. 2Q) and glucose transporter 1 (GLUT1; Fig. 2R) are transport proteins that play major roles in the BBB. While, GLUT1 allows for glucose transport across the BBB^30,31^, P-gp prevents exogenous chemical substances from entering the brain parenchyma^32^. Furthermore, junctional complexes, mainly the tight and adherens junction proteins between HBMECs prevent paracellular diffusion of foreign substances into the brain tissue^33–35^. Through immunofluorescent staining and confocal microscopy, we identified the expression of major tight junction proteins ZO-1 (Fig. 3A) and claudin-5 (Fig. 3B). Adherens junction proteins such as VE-cadherin are anchored by associative protein β-catenin^36^.

Β-catenin signaling stabilizes adherens junction proteins and hence promotes BBB maturation. Even though β- catenin retention at the plasma membrane by cadherins reduces the pool of free β-catenin available for nuclear shuttling to promote expression of genes such as claudin-3^37^, GLUT-1^38^, platelet derived growth factor B^39^, and P-gp^40,41^, it is critical to note that β-catenin shuttling in the nucleus represses claudin-5 gene expression^36^. Thus, β-catenin localization at the plasma membrane is crucial for enhancing BBB maturation through VE-cadherin stabilization and allowing claudin-5 gene expression. We established that β-catenin localized at the plasma membrane in the organoids (Fig. 3C) and VE-cadherin was expressed (Fig. 3D).

### Hypoxia affects junctional protein distribution

One of the primary drivers behind neurologic injury in instances of ischemic stroke and traumatic brain injury is cell hypoxia^42,43^. Mark and Davis showed that hypoxia induces permeability and changes in the organization of tight junctions using a 2D transwell system^44^. Here, we report for the first time physiologic changes of junctional protein distribution in a multicellular 3D brain microvascular equivalent tissue organoid during pathological insult. In order to evaluate the effect of hypoxia on the expression and distribution of tight junction proteins and adherens associated protein, organoids containing all 6 cell types were incubated at 37 °C, 0.1% oxygen and 99.9% nitrogen for 24 hr in an X*vivo* System G300C (BioSpherix, Redfield, NY, USA). Another group of organoids were cultured at normoxic conditions for comparison. Organoids were subsequently fixed and immunostained for claudin-5, ZO-1, β-catenin, and VE-cadherin. Claudin-5, ZO-1, β-catenin and VE-cadherin localization is normal under normoxic conditions (Fig. 3E,G,I and K); however, their localization is disrupted under hypoxic conditions (Fig. 3F–G). Caludin-5, ZO-1, β-catenin and VE-cadherin fluorescence pixels were quantified using ImageJ (National Institute of Health) and were normalized to DAPI fluorescence in each section. ZO-1 and claudin-5 was reduced under hypoxic conditions. The differences, however, were not signficant compared with normoxic conditions (Fig. 3M). We observed a signficant reduction in the relative amount of β-catenin (P = 0.0012) and VE-cadherin (P = 0.0079) under hypoxic conditions compared with normoxic conditions (Fig. 3M).

### BBB charge selectivity assessment by evaluating inorganic mercury toxicity

Evaluation of the organoid model’s application to neurotoxicity was then undertaken through the assessment of inorganic mercury toxicity. Selenium depletion by mercury causes irreversible inhibition of the selenoenzymes that play a critical role in restoring Vitamin C, E and other anti-oxidant molecules back to their reduced forms^45,46^. Vitamin C and E are reducing agents and antioxidants that act as cofactors in reactions catalyzed by Cu^+^-dependent monooxygenases and Fe2^+^-dependent dioxygenases. Mostly, the production of reactive oxygen species is accentuated in brain cells because of the high rate of oxygen consumption in the brain^47^. These high amounts of reactive oxygen species inevitably make brain cells vulnerable to oxidative damage. Thus, brain cells heavily depend on the antioxidant protection provided by selenoenzymes. Brain cell dysfunction and death during prolonged or high mercury exposure is attributed to cellular selenium sequestration by mercury^48^. Selenium is needed for the biosynthesis of critical enzymes such as thioredoxin reductase, an enzyme that prevents and reverses oxidative damage^45,46,48^. Mercury I salts are less toxic than mercury II salts because they are less soluble. Mercury II salts do not cross the BBB despite their high solubility^48^. This makes mercury II salts ideal candidates for validating the charge selectivity function of the BBB in models.

**Figure 4:**
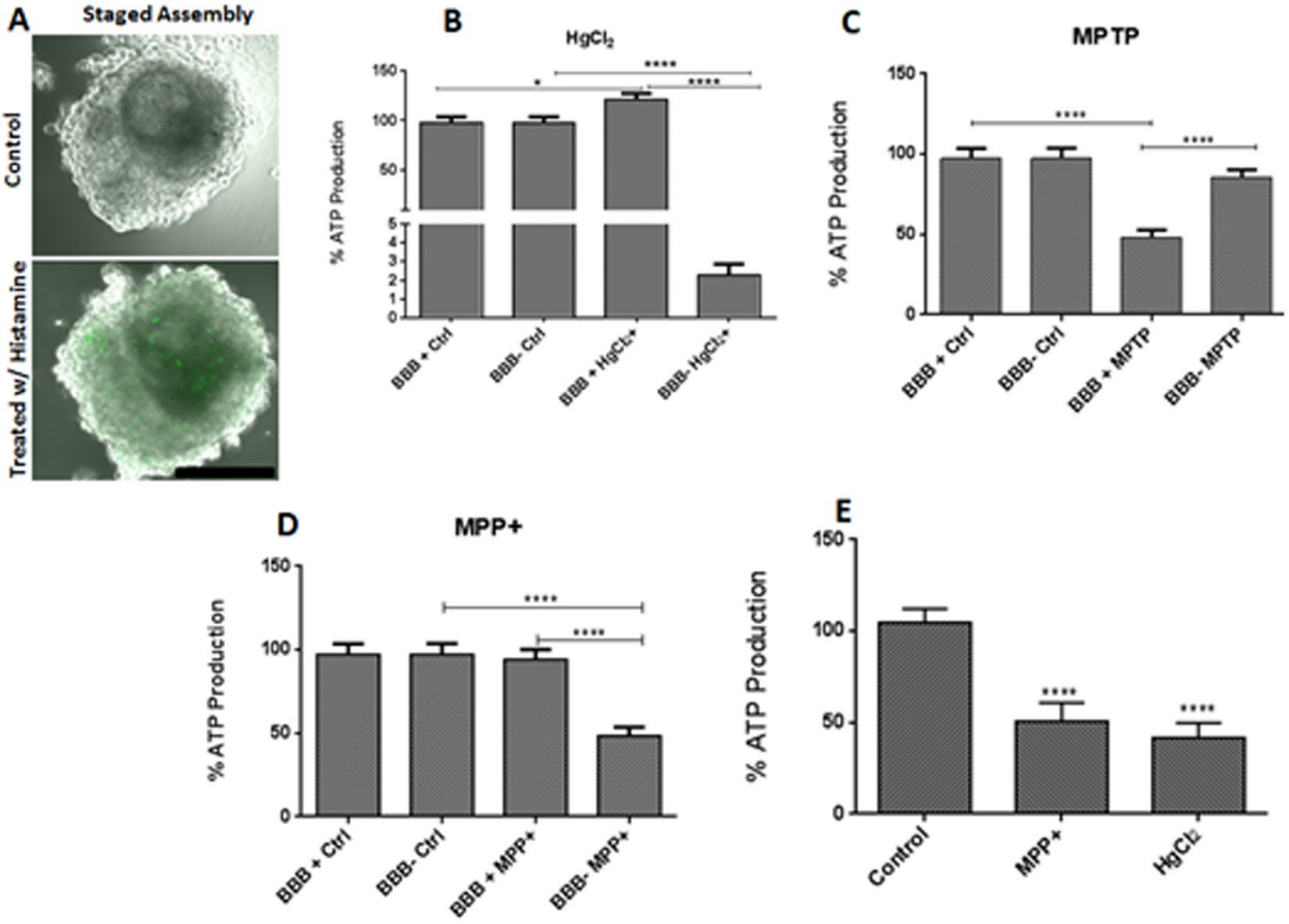
BBB Integrity and selectivity using FITC labeled IgG, Mercury, MPTP and MPP+. (**A**) Shows IgG permeability in untreated (upper panel) and Histamine treated (lower panel) spheroids. (**B**–**D**) Results from 4 separate experiments were pooled and normalized to the ATP production in untreated spheroids. Neuronal (BBB−) spheroids treated with mercury (II) chloride (**B**) exhibited low ATP production. Spheroids consisting of all six cell types (BBB+) that were treated with mercury (II) chloride exhibited higher viability and were statistically significant with respect to their untreated counterparts. In **C**, there was no significant in ATP production in untreated versus MPTP treated BBB− spheroids. MPTP treated BBB+ spheroids exhibited significantly low ATP production compared to the untreated group. MPP+ (**D**) BBB− spheroids exhibited significantly lower ATP production compared to the untreated neuronal spheroids. BBB+ spheroids that were treated with MPP+ exhibited no significantly different cell viability compared to the untreated group. In **E**, BBB+ spheroids were treated with Histamine to allow MPP+ and Mercury ion to penetrate into the organoids. Both MPP+ and mercury (II) chloride demonstrated significantly lower ATP production compared to untreated control. Student T-Test, two tailed hypothesis, *P < 0.05, ****P < 0.0001, with untreated spheroids, n_organoids_ = 64 (**B**–**D**), n_organoids_ = 32 (**E**, experiment only perfomed once). Data represented as mean ± s.e.m.

We constructed organoids containing neuronal cells only (BBB−), as well as organoids containing all 6 cell types (BBB+) using the staged assembly process. Organoids containing only neural cells did not have a BBB, while those containing all 6 cell types promoted the formation of a functional BBB, due in part to the presence of HBMEC, HBVP and HA cells. After completion of staged assembly, organoids were subsequently treated with mercury II chloride for 6 days. Mercury toxicity was evaluated by measuring cell viability through the assessment of ATP production following the CellTiter Luminescent Cell Viability Assay protocol (Promega, Madison, WI. USA). The results showed that BBB− organoids have significantly higher cell death (P < 0.0001) compared to BBB+ organoids (Fig. 4B), demonstrating the low permeability of mercury ions through the BBB. It is worth noting that ATP production significantly increased in BBB+ spheroids treated with mercury II chloride. ATP is required by Na-K ATPase to establish the electrochemical gradient which generates the energy for chloride transport across the cell membrane^49^. This secondary active transport may induce cells to produce more ATP. In order to demonstrate that higher ATP production in BBB+ treated organoids was due to the presence of the BBB, we disrupted the BBB in BBB+ organoids with Histamine. ATP production was significantly lower (P < 0.0001) compared to untreated organoids as shown in Fig. 4E.

### Neuro-toxicity model

A classic murine model of Parkinson’s disease is induced through exogenous administration of a small molecule (173 daltons) pro-drug 1-methyl-4-phenyl-1, 2, 3, 6-tetrahydropyridine (MPTP) into healthy mice^50^. Upon crossing the BBB, MPTP, is metabolized into the toxic positively charged 1-methyl-4-phenylpyridinium (MPP+) by monoamino oxidase B present in glial cells MPP+ selective uptake through the dopamine reuptake pump and subsequent inhibition of complex I by MPP+ causes ATP depletion in the dopaminergic neuron and results in cell death^51–53^. MPTP crosses the BBB due to its lipophilic properties; however, MPP+ is hydrophilic and does not cross the BBB^51,54–56^. Thus, MPTP and MPP+ properties can be employed to assess charge selectivity at the BBB. In order to establish BBB selectivity, we constructed BBB− organoids as well as BBB+ organoids using the staged assembly method. Upon transferring organoids to a long term culture plate on day 4, both the BBB− and BBB+ organoids were treated with MPTP and MPP+ for six days. Cell viability or metabolic activity was assessed by measuring the production of ATP as described above. MPTP did not cause statistically significant cell death in treated compared with untreated BBB− organoids (student t-test, P = 0.13), as MPTP is not toxic to neurons (Fig. 4B), but its metabolite MPP+ caused statistically significant (student t-test, P $<$ 0.0001) cell death in BBB− organoids (C). In contrast, MPTP, caused significant (student t-test, P < 0.0001) reduction in ATP production in BBB+ organoids compared with untreated counterparts (Fig. 4B), while MPP+ did not cause statistically significant (student t-test, P = 0.73) cell death in treated compared to untreated BBB+ organoids, as its hydrophilic properties prevent its passage through the BBB (Fig. 4C). Put together, MPTB caused a reduction of BBB+ organoids compared to BBB− organoids because it traverses the BBB, while MPP+ did not cause lower ATP production in BBB+ organoids. However, MPP+ significantly reduced cell viability in BBB− organoids compared to BBB+ organoids. Furthermore, MPP+ also caused significant (P < 0.0001) reduction in ATP production in BBB+ organoids that were treated with histamine.

## Discussion and Conclusions

*In vitro* BBB models may be critical to the screening and development of novel and robust therapeutics against many neurological insults, and valid one to three cell type models have been described^5,6,57–59^. Additional properties of the BBB can be elucidated by creating organoids with all the cell types present in the brain cortex, in proportions similar to normal tissue. The organoids can serve as a model for the evaluation of interactions between the BBB and adjacent brain tissue, and can provide a platform for understanding the combined capabilities of a novel drug to cross the BBB and its effect on microglia, oligodendrocytes and neurons. Such understanding may be important in designing potential *in vitro* models of neurodegenerative conditions such as amyotrophic lateral sclerosis, multiple sclerosis, or Alzheimer’s disease, and characterizing potential injury models, such as stroke.

The organoid model containing 4 cell types that self-assemble in a manner in which the endothelial cells coat the sphere, and organoids containing 3 cell types that are prepared in a similar method appear to promote the formation of tight junction proteins. The results in organoids containing HBMEC, HA and HBVP show the presence of a functional barrier as demonstrated by low FITC-labeled IgG permeability to the core of the organoids. These organoids also showed a normal response when exposed to histamine, opening the barrier and allowing IgG to cross the barrier. Organoids containing 6 cell types maintained high cell viability for up to 21 days *in vitro*. This may be important in assessing the long-term effect in drug toxicity studies. We also identified expression of P-gp and GLUT1. These proteins play an important role to pump out unwanted chemical substances from the brain tissue and to transport glucose into the brain tissue, respectively. There are many diseases that are implicated in dysfunction and/or other abnormalities in these proteins^50,60,61^ and our 3D model, which mimics the physiological microenvironment, has the potential to be used to evaluate the expression, localization and function of these proteins upon genetic manipulations. We also demonstrated the expression of tight junctions, adherens junctions, and adherens junction-associated proteins; these have been identified to prevent the free paracellular diffusion of substances into the brain parenchyma.

One of the areas that may be important in BBB *in vitro* models is the ability to apply these designs to model adult human disease. Specifically, over 80% of people hospitalized for stroke are adults^62^. Many current *in vitro* models do not accurately mimic adult human physiology. The use of human primary and iPSC derived cells could help narrow the gap towards attaining an ideal model for clinical applications in studies geared towards understanding adult human neurologic diseases. Our data show that hypoxia disrupts the localization of tight and adherens junction proteins, which indicates that our organoid model may be useful in studying ischemia in a physiologically relevant environment.

To evaluate the selectivity of the BBB, we assessed the subsequent effect of mercury ions in the brain parenchyma by evaluating cell viability. Hg^2+^ does not cross the BBB because of its positive charge, which contributes to its hydrophilicity. Our data show that Hg^2+^ that forms when HgCl_2_ dissolves in media only causes significant cell death in neuronal organoids that did not have a BBB compared to organoids that possess a BBB. This indicates that the organoid model containing 6 cell type forms a functional BBB that is charge-selective. Similar results were observed when we used a small molecule pro-drug MPTP (a lipophilic small molecule), which is toxic when enzymatically converted into its metabolite MPP+ in glial cells. MPTP caused cell death or low ATP production in BBB+ organoids. However, when we exposed organoids containing 6 cell types directly to the non-BBB permeable MPP+, there was no significant cell death.

The improvement reported herein warrants this model as a possible alternative to existing *in vitro* models. Further structural characterization to determine the production and proper deposition of extracellular matrix proteins of the BBB is needed. Analysis of effects on individual cell types in the model is also needed to understand cell specific functions which will make the model more applicable to specific applications or the study of various neurologic disorders.

Taken together, the multicellular organoid model forms a functional BBB that is charge selective and shows relevant responses to physiologic conditional changes. This model may be useful not only in drug discovery for novel therapeutics, but also to evaluate the ability of such drug candidates to cross the BBB, and their ensuing sequelae such as general cytotoxicity, glial toxicity and neurotoxicity.

## Methods

### Cells and Culture Conditions

Primary human brain microvascular endothelial cells (Cell Systems, Kirkland, WA) were expanded in plates coated with Attachment factor and were cultured under normal growth condition in complete classic medium supplemented with CultureBoost^TM^ and attachment factor (Cell Systems). Primary human brain microvascular pericytes (HBVP; ScienCell Research Laboratories, Carlsbad, CA) were expanded in plates coated with 15 μg/ml Poly-L-Lysine (ScienCell Research Laboratories) and were cultured under normal growth conditions in pericyte medium (ScienCell Research Laboratories) supplemented with 2% FBS, pericyte growth supplement and penicillin-streptomycin. Human astrocyte (HA) (ScienCell Research Laboratories) were expanded in plates coated with 15 μg/mL Poly-L-Lysine and were cultured under normal growth condition in astrocyte medium (ScienCell Research Laboratories) containing 2% FBS, astrocyte growth supplement, and penicillin-streptomycin. Human iPSC-derived oligodendrocytes progenitor cells (HO; Tempo Bioscience Inc., San Francisco, CA) were propagated in plates coated with 0.2 mg/mL Matrigel (Corning) and were cultured under normal growth conditions in (a) propagation media consisting of DMEM/F12 with HEPES, L- glutamine (2 mM, Life Technologies), non-Essential amino acids (1X Life Technologies), StemPro neural supplement (Invitrogen), PDGF-AA (10 ng/mL, Peprotech), PDGF-AB (10 ng/mL, Peprotech), NT3 (10 ng/mL, Peprotech), Biotin (100 ng/mL, Sigma Aldrich), and cAMP (5 μM/mL Sigma Aldrich) and were then cultured in (b) differentiation media (for 72 hrs prior to organoid formation) consisting of 50:50 DMEM/F12:neuralbasal (Life Technologies), non-essential amino acids (1X Life Technologies), 1x B27 (Life Technologies), L- glutamine (2 mM, Life Technologies), Biotin (100 ng/ml, Sigma Aldrich), PDGF-AA (5 ng/mL, Peprotech), BDNF (10 ng/ml, Peprotech), ascorbic acid (20 μg/mL, Sigma Aldrich), cAMP (1 μM/ml Sigma Aldrich), T3 (200 ng/ml, (Sigma Aldrich). Human iPSC- derived microglia (HM; Tempo Bioscience Inc., San Francisco, CA) were propagated in plates coated with 0.2 mg/ml Matrigel (Corning) and were cultured under normal growth conditions in DMEM/F-12 (Life Technologies), N2 supplement (1x, Life Technologies), essential amino acids (0.5x, Life Technologies) L-glutamine 2 mM, LifeTech), GM-CSF (100 ng/mL, Peprotech), IL-34 (50 ng/mL, Peprotech). Human iPSC- derived neural stem cells (Axol Biosciences Ltd., Cambridge, UK) were plated on plates coated with SureBond (Axol Biosciences) and cultured under normal growth conditions in neural plating-XF medium (Axol Biosciences) 24 hours. The Plating medium was then replaced with neural expansion-XF medium (Axol Biosciences) supplemented with recombinant human FGF2 (20 ng/mL, Axol Biosciences) and recombinant human EGF (20 ng/mL, Axol Biosciences). Once the appropriate number of cells was achieved, the expansion medium was replaced with neural differentiation-XF medium (Axol Biosciences) and cultured for an additional 72 hours to allow for neuronal phenotype differentiation prior to organoid formation. Of note, our initial self-assembly experiment (Fig. 1A) included the use of HCN-2 human cortical neurons (ATCC, American Type Culture Collection, Manassas, VA). These were grown under standard growth conditions in media containing 90% Dulbecco’s modified Eagle’s medium, 4 mM L-glutamine adjusted to contain 1.5 g/L sodium bicarbonate, 4.5 g/L glucose, and 10% fetal bovine serum.

### Organoid Culture

HA, HCN-2, HBMEC, and HBVP were harvested using TrypLE select enzyme (1X Life Technologies), HM and HO were harvested from culture plates with accutase (Life Technologies) and HN were harvested with unlock-XF (Axol Bioscience). To demonstrate self-assembly of organoids containing neuronal cells (HCN-2, Fig. 1A), the cell types were initially labeled using Vybrant® Multi-color Cell Labeling (Life Technologies). They were then mixed in a ratio of 1:1:5:6 – HBMEC, HBVP, HA, HCN-2, respectively. The cells were then cultured using the hanging drop culture method in hanging drop culture plates (InSphero AG, Schlieren, Switzerland). The organoids were allowed to form in media containing EGM-2 media (Lonza). The 3 cell type organoids utilized for initial BBB assessment were cultured in EGM-2 in hanging drop culture plates (InSphero). For organoids containing six cell types, 30% HBMEC, 15% HBVP, 15% HA, 5% HM, 15% HO, and 20% HN were used to make a total of approximately 2000 cells per organoid. Organoids containing HA, HM, HO and HN were allowed to form in 50% astrocyte medium without astrocyte growth supplements and 50% neural maintenance-XF medium under normal growth conditions for 48 hrs in hanging drop culture plates (InSphero). The medium was mixed with heat inactivated FBS (Thermo Fisher) and 10 ng/μL rat tail collagen I (Corning). HBMEC and HBVP were subsequently added to coat the neural-glial organoid. The organoids were cultured under normal growth conditions in 60% neural maintenance-XF medium, 20% astrocyte medium and 20% complete classic medium. The organoids were then allowed to mature further for 48 hr and were dropped into a 96 well plate for long term use. The organoids used in all experiments were between 6–10 days *in vitro* unless specified. Where the single self-assembly method is specified, all cell-types were included in the initial hanging drop and allowed to mature for 96 hrs prior to being dropped into a 96 well plate for long-term culture.

### Cell Viability

Cell viability was evaluated using a Molecular Probe Live-Dead cell imaging system (Invitrogen). Human brain organoids were harvested for cell viability analysis at days 4, 5, 7, 10 and 21. Organoids were incubated at room temperature for 10 minutes in DPBS containing 2 μM calcein AM (Invitrogen, green- live cells) and 4 μM ethidium homodimer-1 (Invitrogen, red - dead cells). After washing once with DPBS, the organoids were then imaged using the Olympus Fluoview Fv10i (Olympus, Tokyo, Japan) laser scanning confocal microscope. The images obtained were then quantified using ImageJ to determine cell viability percentages.

### Immunohistochemistry

Organoids were collected into 1.7 ml Eppendorf tubes (Corning Inc). After aspirating the media, the organoids were fixed in 4% formaldehyde (Polysciences Inc, Warrington, PA) for 15 minutes at 4 °C and were washed 3 times with cold PBS. The organoids were permeabilized with 0.1% Tween-20 in PBS for 10 minutes at 4 °C and washed 3 times. Organoids were exposed to Protein Block (Dako Group, Troy, MI) for 1 hr at RT, and organoids incubated at 4 °C overnight in Antibody Diluent (Dako) solution containing primary antibodies –anti-O4 (a marker for OPC, 1:200, RD Systems), anti-Myelin Binding Protein (a marker for mature oligodendrocytes, 1:250, Millipore, Billerica, MA), anti-PDGF-receptor-b [CD140b] (a marker for pericytes, 1:500, Millipore), anti-GFAP (a marker for astrocytes, 1:500, Millipore), anti-CD31 (a marker for endothelial cells, 1:1000, Abcam), anti-Iba1 (a marker for microglia 1:500, Abcam), anti-beta Tubulin (a marker for neurons 1:1000, Abcam), anti-beta Catenin (1:500, Abcam), anti-GLUT1 (1:500, Abcam), anti-VGLUT2 (1:500, Abcam), anti-MDR-1 (1:500, Millipore), anti-ZO-1 (1:1000, Millipore), anti-Claudin-5 (1:500, Millipore), and anti-Tyrosine Hydroxylase (1:300, Millipore). Organoids were subsequently washed 3 times and incubated with AF488 Goat anti-Mouse IgG (1:1000, Life Technologies), AF594 Goat anti-Rabbit IgG (1:1000, Life Technologies), AF488A Goat anti-Chicken IgG (1:500, Biotium) in Antibody diluent (Dako) overnight at 4 °C. Nuclear staining was perfomed by incubating the organoids with DAPI (1:1000) in PBS for 10 minutes. The organoids were washed and imaged using the Olympus Fluoview Fv10i (Olympus) laser scanning confocal microscope. At least three randomly selected organoids were imaged for each stain.

### Hypoxia

On day 6, a 96 well plate containing organoids was cultured in media (60% neuronal maintenance media, 20% endothelial growth media, and 20% astrocyte growth media) at 37 °C, 0.1%O_2_, 99.9%N for 24 hr in an X*vivo* System G300C (BioSpherix, Redfield, NY, USA). 16 randomly selected organoids grown under normoxic conditions and 16 randomly selected organoids grown in hypoxic condition were immediately fixed in 4% PFA and subsequently prepared for immunofluorescent staining for ZO-1 and beta catenin. This experiment was repeated twice.

### Assessement of FITC labeled IgG Permeability

One group of organoids was incubated in media at normal growth conditions with FITC Conjugate IgG (1:200, Millipore) for 30 minutes. A control group was treated with 4.5 mM Histamine 15 minutes prior to incubating with FITC labeled IgG. The organoids were washed three times before imaging with the Olympus Fluoview Fv10i (Olympus) laser scanning confocal microscope. ImageJ was used to quantify fluorescent intensity.

### Neurotoxicity

Organoids were treated with growth media (60% neuronal maintenance media, 20% endothelial growth media, and 20% astrocyte growth media) containing MPTP, MPP+, HgCl_2_ (10 μM, Sigma). The control group was cultured in media without drug or compound. Media was replaced with fresh media change containing MPTP, MPP+, HgCl_2_ every 48 hrs for six days. Media was replaced with fresh media containing histamine and MPP+ or HgCl_2_ every 48 hrs for experiments in Fig. 4E. Neurotoxicity and glial toxicity was assessed by measuring ATP production on day 6.

### ATP Production

The organoids were transfered to an opaque-walled 96 well plate. Media (100 μL) was added to wells. CellTiter-Glo Substrate and CellTiter-Glo Buffer (Promega Life Sciences, Madison, Wisconsin) were mixed as per manufacturer’s instructions to make the CellTiter-Glo Reagent. The reagent (100 μL) was then added to 100 μL of medium containing organoids. The contents were then mixed well on an orbital shaker to induce cell lysis. The luminescent signal was stabilized by incubating the plate at room temperature for 10 minutes. Luminescence was measured using a microplate luminometer (Veritas, Mountain View, CA). Background luminescence was then subtracted from the samples.

### Data availability statement

All data generated or analysed during this study are included in this published article.
